# *Salmonella* in Chicken Meat: Consumption, Outbreaks, Characteristics, Current Control Methods and the Potential of Bacteriophage Use

**DOI:** 10.3390/foods10081742

**Published:** 2021-07-28

**Authors:** Kirsten Wessels, Diane Rip, Pieter Gouws

**Affiliations:** Centre for Food Safety, Department of Food Science, Stellenbosch University, Stellenbosch 7600, South Africa; 20288018@sun.ac.za (K.W.); dirip@sun.ac.za (D.R.)

**Keywords:** bacteriophage, *Salmonella*, chicken, poultry

## Abstract

The control of *Salmonella* in chicken processing plants is an ongoing challenge for many factories around the globe, especially with the increasing demand for poultry escalating processing throughputs. Foodborne outbreaks due to *Salmonella* still pose a prominent risk to public health. As chicken meat is a good reservoir for *Salmonella*, it is important for chicken processing plants to continuously optimize methods to reduce the incidence of *Salmonella* on their products. Current methods include the use of chemical antimicrobials such as chlorine-containing compounds and organic acids. However, these current methods are decreasing in popularity due to the rising rate of *Salmonella* resistance, coupled with the challenge of preserving the sensory properties of the meat, along with the increasing stringency of antimicrobial use. Bacteriophages are becoming more appealing to integrate into the large-scale hurdle concept. A few factors need to be considered for successful implementation, such as legislation, and application volumes and concentrations. Overall, bacteriophages show great potential because of their host specificity, guaranteeing an alternative outcome to the selective pressure for resistant traits placed by chemicals on whole microbial communities.

## 1. Global Trends in Poultry Consumption

Poultry largely outnumbers humans with approximately one person for every three birds [1]. Meat and eggs produced from poultry are consumed across numerous cultures and are among the most efficient forms of protein [1,2]. In 2016, the global livestock environmental assessment model (GLEAM) generated by the Food and Agriculture Organization of the United Nations [3] approximated egg production to be 73 million tons and meat production to be 100 million tons. These numbers are constantly increasing due to population growth, escalating incomes and urbanization [1,4,5]. Demand for poultry is increasing not only in developing countries but also developed countries [5,6]. The demand is met because chickens are intensively produced; chickens rapidly reach a sufficient size and are then slaughtered and processed through highly automated systems that allow for rapid throughputs [6]. 

The shift from free range farming towards intensive practices has allowed for tremendous growth in the supply of poultry as a protein source [6]. Intensive practices have utilized various breeding techniques, feed manipulation and antibiotic administration to optimize size, growth, and desirable attributes [1,7]. 

Animal sourced protein provides various micronutrients [2] that are challenging to acquire in sufficient quantities from plant based protein, such as vitamins A and B, zinc, iron, and calcium [1]. Poultry, specifically, is cheap, a high quality source of protein and has very few negative associations with religious beliefs, and is therefore often the animal protein of choice in developing countries [1,8]. 

In a study conducted by Zeng et al. [9], where trends in meat consumption were tracked and analyzed in American adults from 1999 to 2016, it was found that chicken consumption increased from approximately 250 g per week in 2000, to 300 g per week in 2016. Conversely, the consumption of turkey remained relatively constant. Furthermore, in Kuwait, average poultry meat consumption per capita from 2004 to 2016 was a whopping 64.4 kg/year (approximately 1.2 kg/week) [10]. Another country showing substantial growth is Brazil; Brazil is the country with the largest export rate of poultry meat and the second highest poultry meat producer globally, making it a top competitor with China and the US [11]. This increased preference and, consequently, production could be due to a couple of factors: firstly, because the price of red meat has increased while the price of chicken has remained constant, and secondly, many health concerns have been associated with red meat which, thus, have created the perception of chicken being a healthier and leaner option [9,12]. 

Another trend which is affecting the supply of poultry is ready to eat (RTE) meals. This includes snack foods, take away meals and dining out. This manner of consumption is becoming more popular and is seen as more convenient than preparing a meal in the home [6,13]. 

With this increasing demand, there are many consumers that are becoming increasingly aware of quality and are now purchasing products with the consideration of food safety, environmental impact, and animal welfare [6,14]. This forces the industry to keep up with the increasing sophistication and refining of food technology [14]. The poultry industry on a global scale is significantly influenced by these four areas of pressure in society, namely, food security, the economy, environmental impact and food safety [1]. These four dimensions are responsible for the delicate balance that the poultry industry continuously struggles to satisfy with the rapidly increasing demand. 

## 2. Poultry Associated Outbreaks

Globally, poultry is the second highest in terms of meat consumption and is predicted to increase more rapidly than any other meat type. This makes poultry a predominant source of foodborne illness [15]. There are a few pathogens strongly associated with foodborne outbreaks in poultry, one of the most common being *Salmonella* [15,16,17].

The United States Centers for Disease Control and Prevention (CDC) facilitate a note-worthy system whereby clinics collect samples of bacteria isolated from ill patients and submit them to public laboratories. The laboratories then identify the subtypes of the samples using pulsed field gel electrophoresis (PFGE) [18]. These subtypes are then made available on a database (PuleNet) which is accessible nationwide to various organizations which can identify sources of illness caused by a common PFGE subtype [18]. Furthermore, PulseNet also makes use of whole genome sequencing (WGS) which determines the order of bases (genetic fingerprint) in a DNA sequence in a single laboratory procedure [19]. WGS supplies more intricate information to assist in identifying outbreaks: PFGE compares 15–30 bands, whereas WGS identifies millions of bands, making it easier to distinguish if the bacteria are in fact the same [19]. In 2017, the CDC identified poultry products (turkey and chicken) as the dominant source of *Salmonella* infections resulting in illness (Table 1). 

In an analysis conducted by Chai et al. [15], whereby 1114 outbreaks from 1998 to 2012 in the United States were investigated and analyzed according to a strict criterion, 279 of the total 1114 outbreaks (25%) were linked to poultry. Of the 279 outbreaks, 149 could be traced back to a confirmed pathogen. Out of these 149 outbreaks, approximately 43% was due to *Salmonella*, 26% *Clostridium perfringens*, 7% norovirus, 7% *Campylobacter*, 5% *Staphylococcus aureus*, 3% *Bacillus cereus* and a further 3% was due to *Listeria monocytogenes* [15]. 

Furthermore, in the analysis, the outbreaks associated with *C. perfringens*, *S. aureus* and *B. cereus* were due to errors in food-handling, while the *Salmonella* outbreaks were predominantly due to contamination prior to cooking or insufficient cooking [15]. 

Dominguez et al. [21] analyzed outbreaks in Catalonia, Spain from 1990 to 2003. Of the 1652 outbreaks, 871 (52%) were due to *Salmonella.* Of these 871 outbreaks, there were more than 1500 people who needed hospital care and there was a total of four deaths [21]. Half of the outbreaks caused by *Salmonella* were traced back to eggs (food with raw or partially cooked eggs). In the same study conducted by Dominguez et al. [21], 207 (12.5%) of the 1652 outbreaks were due to *C. perfringens*, *norovirus*, or *S. aureus*.

The most common foodborne disease caused by poultry meat is salmonellosis, named after the causative bacterial agent *Salmonella* [22,23]. Many preventative and control measures have been developed and implemented in efforts to control *Salmonella* on poultry products, however, resistant strains have rapidly emerged, causing outbreaks despite extensive quality management systems [17,24]. Salmonellosis is caused by serotypes of *Salmonella* other than *Salmonella enterica* serovar Typhimurium and *Salmonella* Paratyphi; the common serotype responsible for most outbreaks related to poultry is the *Salmonella enterica* serotype Enteritidis [17]. The difference between these will be further explained in Section 3. Salmonellosis involves symptoms such as fever, diarrhea, and severe cramp, with an incubation period of up to 72 h after consumption [17]. According to Majowicz et al. [16], *Salmonella* is responsible for 93 billion cases of illness and approximately 155,000 fatalities globally each year.

Jackson et al. [25] analyzed 1491 outbreaks due to *Salmonella* recorded by The Foodborne Disease Outbreak Surveillance System (FDOSS) in the United States. The outbreaks took place between 1998 and 2008; of the 1491 outbreaks, approximately 400 were caused by a known serotype and could be assigned to a food. Of the 400 outbreaks, 144 (36%) were due to *S. enteritidis* and 24 (6%) were due to *S*. *heidelberg*—these outbreaks were traced back to eggs [25]. A further 58 outbreaks were due to *S.* Typhimurium and were traced back to chicken [25].

Canada noted a total of 18 outbreaks and nearly 600 WGS confirmed cases of *Salmonella* infections from 2015 to 2019 that could be traced back to frozen raw breaded chicken products [26]. While the European Food Safety Authority (EFSA) [27] recorded 193 cases (20% were hospitalized) of *S. enteritidis* between 2018 and 2020—2 were in Denmark, 4 in Finland, 6 in Germany, 12 in Ireland, 3 in the Netherlands, 5 in Poland, 6 in Sweden, 33 in France and the other 122 in England. This outbreak was traced back to five production batches of non-RTE breaded poultry products.

Kenny et al. [28] analyzed 10 reported cases of *S. typhi* in South Australia that were recorded within a period of four weeks of each other. Data of the foods eaten for the five days prior to the symptoms was collected—chicken nuggets appeared frequently which led to a case study that investigated whether the consumption of the chicken nuggets was linked to the onset of the illness. Controls were included in the case study, thorough interviews were conducted and, finally, the *S*. *typhi* strain isolated from the brand of chicken nuggets from a packet found in the home of one of the cases was found to be common with nine out of the reported ten cases of illness [28]. The chicken nuggets that were responsible were flash fried but were still classified as a product that needed to be cooked. More recently Australia has continued to see an increase in cases of human salmonellosis (approximately 70 cases per 1,000,000) [29].

This, once again, reiterates the necessity for clear labelling and sufficient cooking to exclude the potential of infections due to *Salmonella* from poultry, and it also highlights the need for continuous efforts to control *Salmonella* contamination in chicken [30]. Furthermore, to prevent contamination of food products it is important to implement good hygiene practices for all handling and processing of food, as seen in Table 2 for processing of chicken. 

## 3. Salmonella 

As previously mentioned, foodborne outbreaks pose many risks, both in terms of health and economic loss. The pathogen of particular emphasis and concern in poultry is *Salmonella* [32,33,34]. The United States, alone, spends approximately 11.588 billion dollars on collateral damage and improving prevention methods for *Salmonella* infections originating from poultry products annually, while the EU’s estimated costs are more than €3 billion a year [33,35]. *Salmonella* has been pinpointed as the source of many cases of food poisoning as well other severe health defects over the last century [32,36]. The continual outbreaks due to *Salmonella* make this resilient genus and its characteristics a focused point of research for many health and science professionals despite an existing abundance of information [33]. The survival of *Salmonella* can be accredited to its resistance-development rates being more rapid than that of other pathogenic bacteria placed under the same preventative pressures [36,37]. Managing an organism that is changing incessantly requires an in depth understanding of its characteristics and what the outward expression from these characteristics may imply upon human consumption [32]. 

### 3.1. General Characteristics

The genus of *Salmonella*, under the family of *Enterobacteriaceae*, are rod-shaped (approximately 2 μm in size), motile (due to presence of peritrichous flagella), glucose-fermenting, Gram-negative, facultative anaerobes that do not form spores [38,39,40]. *Salmonella* can commonly be found on dairy products, meat products (especially raw poultry) and fresh produce [36]. The various parameters and conditions in which *Salmonella* can survive are given in Table 3. As *Salmonella* is not a spore-former, it can be destroyed easily with heat, particularly in food products with high water activities [32]. Forysthe [34] tells us that a temperature–time combination of 15–20 min at 60 °C should be sufficient to ensure the death of all *Salmonella* present in the food product, and Bell and Kyriakides [41] also assure us that growth of most serotypes of *Salmonella* will be inhibited below 7 °C and a pH of 4.5. 

### 3.2. Salmonella Serovars 

The *Salmonella* genus is further divided into two species, namely, *Salmonella enterica* (*S*. *enterica*) and *S. bongori* [42]. Serovars of *Salmonella* can be grouped by their O (somatic), Vi and H (flagellar) antigen combination; O antigens being lipopolysaccharides of the outer membrane, Vi antigens being the sugar composition on the capsid, and the H antigens being the sugar combination found on the flagella [43]. This method of identification is responsible for the quarter of a million serovars widely recognized so far, with the majority of the serovars from *S*. *enterica*, a number that is increasing annually [42]. Furthermore, serovars can also be identified using phage sensitivity testing, whereby the *Salmonella* is treated with specific, known bacteriophages and the resulting lytic activity reveals which serotype of *Salmonella* is present due to the range of host specificity of the bacteriophage [44]. 

The system of identifying and categorizing *Salmonella* can be confusing due to more than 250,000 known serovars [40,43], Forsythe [34] simplifies this, and, rather, emphasizes the importance of three different types of *Salmonella* with regards to human health: non-typhoid *Salmonella, Salmonella typhi* (*S. typhi*) and *Salmonella paratyphi* (*S. paratyphi*). 

Non-typhoid *Salmonella* is distinguished by an incubation period of 6–72 h after consumption, causing symptoms such as diarrhea, blood in the stools, consequent dehydration, fever, vomiting, weakness, and abdominal pain [40]. Conversely, *S*. *typhi* and *S*. *paratyphi* have an incubation period of 1–4 weeks, causing symptoms that are like typhoid, such as headaches, fever, body weakness and aches, constipation, or diarrhea [34]. 

Various food properties influence the infectious dose of different serotypes of *Salmonella*. For example, in foods that have a higher fat content, the bacterial cells are protected and thus fewer than 100 cells may cause illness [41]. Thus, a standard level of detection in RTE foods had to be established that ensured that food safety would be maintained despite the serotype. Thus, it was determined that there should be less than one cell of *Salmonella* per 25 g of a RTE food sample [45]. 

## 4. Treatment of *Salmonella* in the Slaughter Setting 

Despite stringent measures and efforts in rearing chickens in a way that seeks to eliminate *Salmonella* from the hatchery level—such as good hygiene practices, isolating infected flocks and the use of specialized feed—the safe passage of poultry from farm to fork remains under scrutiny due to contaminated poultry meat continuously having the largest negative impact on public health. Thus, it is important that the processors of poultry meat utilize existing, new, or additional measures to assist in the prevention of *Salmonella* [46,47,48]. In the United States, poultry processing facilities have had to employ a criterion established by the United States Department of Agriculture’s Food Safety and Inspection Service (USDA-FSIS) whereby for every 51 samples collected, less than 7.5% of them should be *Salmonella* positive [49]. 

Some of the measures employed by poultry processors include a postchilling immersion tank with various antimicrobials as well as spray applications, also with various antimicrobials. The combination of these methods/addition of these methods to existing preventative measures create a “hurdle concept” in the processing plant for the elimination of *Salmonella* [47,48]. Some of these antimicrobials and their respective applications can be seen in Table 4.

### 4.1. Chlorine

Awareness surrounding the use of chlorine as a disinfectant came about as early 1868, when chlorine was found to be a core chemical in curing puerperal fever [51]. Around 1988, however, it was discovered that compounds containing chlorine also have oxidative properties [51]. There are several different chlorine-containing compounds that are used to kill bacteria and are often a popular choice due to a combination of affordability, easy implementation, and a high efficacy [52,53].

When chlorine is added to water, it reacts with the hydrogen and oxygen of the water molecule, resulting in the formation of hydrochloric acid (HCl) and hypochlorous acid (HOCl). HOCl further undergoes dissociation to form hypochlorite (OCl^−^) and hydrogen (H^+^) ions. Both HOCl and OCl^−^ account for the “free chlorine” in a solution and are the main compounds behind the antimicrobial action from the addition of chlorine [54,55]. The mode of action of free chlorine can be divided into three steps: first, the free chlorine compounds disrupt the bacterial cell wall, which causes bacterial DNA to leach out of the cell (Britton, 2005). After this, the free chlorine proceeds to interact with the cell nucleic material and enzymes which inhibits their normal processes [56]. Lastly, the free chlorine may also interrupt transport and respiratory mechanisms in the cell, which negatively effects the cells overall viability [56]. 

Slow release chlorine dioxide (SRCD) is often used in the processing of poultry to decrease *Salmonella* on carcasses [57]. The use of SRCD as an antimicrobial is necessary because, despite efforts that ensure the number of live birds that have *Salmonella* are low, there is an inevitable spread of *Salmonella* due to the mechanical action of the plucking machine, as well as the damage to innards during the evisceration step. Furthermore, the level of contamination of carcasses that end up in the supermarket is something which is strongly correlated to the amount of cross-contamination which occurs during the processing [58]. A 10% SRCD carcass rinse used in combination with a 50 ppm chlorine solution in the spin chilling step has shown to reduce *Salmonella* by more than 80% [58]. 

Despite often being used in combination during processing, SRCD is preferred over chlorine as chlorine may form carcinogenic chlorinated hydrocarbons in the presence of organic matter [59].

Byun et al. [53] carried out a study that investigated the use of chlorine-containing compounds against *S. enteritidis* biofilms in the presence of organic matter. The use of chlorine dioxide (100 μg/mL) reduced counts by up to 1.33 log CFU/cm^2^. While Chousalkar et al. [60] found that acidified sodium chlorite had a high efficacy in reducing all *Salmonella enterica* serovars on chicken carcasses at various temperatures.

Roller et al. [61] and Sun et al. [62] describe the mode of bacterial inactivation using chlorine dioxide as one which disrupts the dehydrogenase enzymes in the bacterial cell. This consequently inhibits protein synthesis to a certain extent, whereby the extent of protein synthesis inhibition was found to be strongly related to the initial concentration of chlorine dioxide added [57,61].

SRCD—as well as other chlorine-containing antimicrobials—are a popular choice for disinfection due to chlorine’s versatility, relatively low cost and effectiveness in reducing bacterial populations [52,58]. These chemicals, although shown to have a good efficacy, are not permitted in the EU [63].

#### Limitations of Chlorine-Containing Antimicrobials 

Legislation is becoming more stringent on the use of chlorine-containing compounds for use in the food industry due the formation of harmful byproducts among other potential hazards [53]. The use of chlorine-containing antimicrobials in the poultry processing setting for treatment of Salmonella spp. has become an exceptional cause for concern. Logue et al. [64] and Shah et al. [65] argue that while chlorine may significantly reduce microbial populations, it can also promote the selection for chlorine-resistant strains of Salmonella. Although, in the short term, safe levels are essentially achieved via chlorination, it may present larger challenges for future treatment of microbes with a chlorine-resistance factor [64,65]. 

A prime example of this is presented in a study conducted by Mokgatla et al. [66], where the resistance of *Salmonella* to hypochlorous acid (HOCl) was investigated. The *Salmonella* spp. investigated in this study were isolated from various processing steps in a poultry abattoir, these were then added to a Tryptone Soya Broth with HOCl at 72 ppm and placed in a shaking incubator at 30 °C. The turbidity of the solution was measured at 660 nm at successive 20 min intervals thereafter. It was found that *Salmonella* spp. isolated after the scalding step were resistant to the addition of the 72 ppm HOCl [66]. 

In a separate study which then investigated the mode of action of HOCl resistance in *Salmonella,* it was found that the HOCl-resistant strains would produce catalase in response to treatment with HOCl [67]. Furthermore, the HOCl resistant strains would also decrease dehydrogenase activity which led to decreased concentrations of oxygen and hydroxyl radicals, the compounds predominantly responsible for the antimicrobial properties of HOCl [67]. 

*Salmonella* has a high resistance rate [37,68] and certain isolates will overcome chemical antimicrobials to an extent such that the chemical compounds may even have a selective consequence that allows for exponential growth of *Salmonella* [66].

### 4.2. Organic Acids

The use of organic acids is also a popular choice of antimicrobial in meat processing plants due to the combination of high efficacy and low cost, as well as the ease of use [69]. Furthermore, the United States Food and Drug Administration (FDA) have designated organic acids the “Generally Recognized as Safe” (GRAS) title for use in meat processing [69,70]. 

Organic acids are commonly used as part of the hurdle concept in preventing growth of *Salmonella* in the processing environment [69]. Organic acids inhibit bacterial growth by lowering the pH of the meat product to a pH equal to—or less than—the pKa of the organic acid [71,72,73]. Essentially, the organic acids inhibit the bacterial cell by causing an accumulation of anions in the bacterial cytoplasm which negatively effects the bacterial cell’s proton motive force (PMF) and, thus, the cell’s ability to maintain an optimum pH [69,71]. This consequently disrupts the internal environment of the cell and inhibits DNA synthesis as well as normal enzymatic activity and cell reproduction [71,72,73]. 

Madushanka et al. [72] explored the efficacy of various organic acids on chicken meat contaminated with *S. typhimurium*. Lactic acid (1% solution) achieved a 66% reduction in CFU/g, while acetic acid (1%) and citric acid (1%) showed a 55% and 51% reduction, respectively. 

Fernández et al. [74] dipped *Salmonella*-contaminated chicken breast in 3% solutions lactic, malic and fumaric acid. It was found that fumaric acid had the highest efficacy (up to 2.22 log CFU/g reduction) but affected the sensory properties of the chicken breast the most. While lactic and malic acid showed reductions of 1.30 log CFU/g and 1.55 log CFU/g, respectively, but did not have any detrimental effects on the sensory properties compared to the control samples. 

Radkowksi et al. [75] tested the efficacy of various concentrations (2% and 5%) of succinic acid on the reduction of *Salmonella* on broiler chicken breast samples. A 2% succinic acid solution achieved a reduction of up to 1.47 log CFU/g while 5% solution achieved up to 3.2 log CFU/g reduction. 

Consansu and Ayhan [76] performed an experiment to determine the effects of lactic and acetic acid on *S. enteridis* on chicken products. Chicken legs and breasts were inoculated with *S.* *enteridis* and were then treated with various concentrations of lactic acid or acetic acid. Some of the samples were allowed to stand for 10 min and were then tested, while others were then packaged and stored at refrigeration temperature for 10 days or were otherwise frozen and stored for six months. Lactic acid achieved the highest reduction in both leg and breast samples (up to 1.72 log reduction). Overall, it was found that both acids were mostly effective in reducing S. Enteridis. However, despite reduction, remaining *S.* *enteridis* were able to survive refrigeration and freezing temperature. This highlights how organic acids should be used in conjunction with other methods to ensure sufficient reduction is achieved [76,77].

#### Limitations of Organic Acids

The bactericidal activity of organic acids is largely dependent on contact time, temperature, the concentration of the acid used or what it is used in combination with [78]. This may be problematic, especially with the high rate of *Salmonella* resistance and the constant need to ensure sufficient kill is achieved by the specific method used [37,79]. 

Despite the relatively high efficacy frequently achieved by organic acids, there is also the risk of adding organic acids at a level and temperature which may affect the sensory properties of the meat [70,78,79]. In a study conducted by Bilgili et al. (1998), the effect of various organic acids on broiler skin color was investigated; it was found that all acids (citric, lactic, malic, mandelic and tartaric), except for propionic, decreased the lightness of the broiler skin as the concentration of each acid increased. The skin/carcass appearance is important for consumer perception and acceptance and, thus, is very important to consider when selecting an organic acid as an antimicrobial [78,80]. As well as undesired colors and textures, organic acids can also cause off flavors and, despite a high efficacy, possess a delicate balance between the ability to compromise desirable sensory properties in exchange for reduced microbial populations [80].

## 5. Bacteriophages

### 5.1. Background

Antibiotic resistance has compromised the effectiveness of antibiotics as a treatment against infections [81,82]. Antibiotic resistance is caused by the misuse of antibiotics in the treatment of an illness; this results in the targeted bacteria no longer being sensitive to the antibiotic for which it was created [83]. In the US, an annual estimate of approximately 23 × 10^6^ kg of antibiotics are used, of which 50% are administered to humans while the other 50% are used for livestock in disease prevention/treatment [82]. Due to the rising numbers of organisms resistant to antibiotics, it is essential that more than one treatment should be available for various illnesses to avoid a situation like that before the existence of antibiotics, when there was a high death rate due to common infections [83]. Antimicrobial resistance is also on the rise where surface and cleaning antimicrobials are no longer able to eliminate the bacteria of concern, thus, we face a large scale resistance problem which requires urgent attention and alternatives, and a possible solution is bacteriophages [81,82,84].

The discovery of the bacteriophage phenomenon is largely debatable: Ernest Hankin in 1896 “first” suggested that there was an invisible, inexplicable antibacterial activity of *Vibrio cholerae* that he noticed in the rivers of India [85,86]. He further suggested that whatever was responsible for this antibacterial activity was small enough to pass through porcelain filters [85]. Eventually, Frederick Twort, some 20 years later, suggested that Hankin’s findings could have been a virus, and, finally, two years after this, Felix d’Herelle “officially” classified this virus as a bacteriophage [87,88].

Phages naturally exist in abundance all around us: in fresh water it is suggested that there are approximately 10^9^ phages/mL while marine environments may have up to 10^7^ phages/mL [89]. Fermented foods, fresh vegetables, topsoil and even delicatessen foods have been found to be good sources of phages too, meaning that humans are constantly exposed to—or are consuming—phages [89]. 

Bacteriophages (phages) are known as predators of bacteria; phages are essentially viruses which infect and subsequently cause bacterial cell death. Phages attach themselves to specific receptor sites on the bacterial cell wall, meaning that phages will only infect a specific range of bacteria while any other present cells or organisms will be unaffected [90,91]. Hence why phage consumption by humans has no adverse effects and can be given the GRAS status [89,91]. After attachment to the bacterial cell wall, the phage injects its genetic material into the bacterial host which causes the genes of the phage to be expressed and ultimately causes the bacterial cell to die [92]. 

Depending on whether the bacteriophage is virulent or temperate, one of two events may occur after bacterial cell infection [89,90].

Virulent phages (also known as strictly lytic) are phages that cannot incorporate their genetic material into the bacterial chromosome to create lysogens, this means that after infection, virulent phages will always initiate replication within the host, progeny and then lysis (cell death) of the bacterial cell [89,92,93]. 

Temperate phages (also known as lysogenic), on the other hand, may cause progeny but not kill the bacterial host cell or may integrate some of the phage genetic material into that of the hosts. This results in the replication of the bacterial DNA along with the phage DNA which may result in modifications of the host characteristics, which could lead to host resistance. Alternatively, phage genomes introduced into that of the bacterial genomes may undergo recombination and lead to undesirable changes in the phage genome [94,95,96]. 

Thus, it is preferable to use phages that are virulent (lytic), rather than temperate, for phage therapy because destruction of the bacterial host is rapid and there is minimal chance of interactions with the host genome [94,96,97]. 

### 5.2. Phage Application to Reduce Salmonella on Food and Poultry Products

Most lytic phages used for biocontrol on food products are generally isolated from the environment and not genetically modified. Due to the host specificity of the phages, other beneficial microflora present in food remains intact [91,98,99]. Phage solutions are predominantly water based, contain low concentrations of salt, and are considered environmentally friendly, appealing to many of the consumers’ demands [91]. Furthermore, phages have very little/no effect on the organoleptic properties of food [81], while having a high efficacy in microbial reduction [91,100,101].

Modi et al. [102] investigated the survival of *S*. *enteritidis* during cheddar cheese storage in the presence of SJ2 phages. Both the raw and pasteurized milk were inoculated with *S*. *enteritidis* and SJ2 phages. Of the resulting cheeses, it was found that those made from raw/pasteurized milk containing phages showed up to a 2-log unit reduction of *S*. *enteritidtis* after 99 days. Conversely, those made from raw/pasteurized milk without phages showed an increase in *S. enteritidis* of up to 1 log unit [102]. When comparing raw versus pasteurized milk, it was found that there was less *S. entertidis* after 24 h in the phage-containing pasteurized milk cheese versus phage-containing raw milk cheese. After 99 days, the phage-containing raw milk cheese had approximately 50 CFU/g *S. enteritidis* while the phage-containing pasteurized milk cheese had no counts of *S*. *enteritidis* after just 89 days. This study highlights the effectiveness of phage to reduce the survival of *S. enteritidis* in cheese [102], as well as the necessity for the phages to be used in addition to other microbial control methods [100]. 

Looking at chicken specifically, Goode et al. [103] aseptically cut 60 cm^2^ squares of chicken and artificially contaminated them with *Salmonella* strains that showed resistance to nalidixic acid. The strains were cultured overnight in Luria–Bertani (LB) broth in a shaking incubator at 37 °C. The 60 cm^2^ pieces of chicken were then artificially contaminated with *S. enteritidis* using a pipette and glass hockey stick. The 60 cm^2^ chicken piece was then treated with *Salmonella* typing phage 12 at 10^3^ PFU/cm^2^ and stored at 4 °C. Swabs were taken before phage treatment, after 24 h and 48 h. Bacterial numbers fell by 2 log units after 48 h, and it was found that an increase in phage concentration up to 10^7^ PFU/cm^2^ eliminated the strains which showed strong resistance to nalidixic acid [103]. 

Hungaro et al. [104] carried out a study like that of Goode et al. [103], except the efficacy of a bacteriophage cocktail against *S*. *enteritidis* was tested versus conventional chemical agents. The use of bacteriophage resulted in a 1 log unit reduction (Table 5) after 30 min, while lactic acid caused a 0.8 log unit reduction after 90 s. The results are highly comparable, however, chemical and physical treatments above certain levels have an adverse effect on the organoleptic properties of the carcass and, thus, the biological intervention of bacteriophages is the more appealing option [101]. 

Sukumaran et al. [105], furthermore, highlight the high efficacy of chlorine immersion in the spin chilling step but also how this efficacy is reduced due to the large amounts of organic matter in the spin chiller solution. Sukumaran et al. [105] carried on and investigated the potential of using bacteriophage in sequence and in combination with various chemical methods, and how this may affect phage stability and overall ability to reduce *Salmonella*. Firstly, phage stability in peracetic acid (PAA), cetylpyridinium chloride (CPC), lauric arginate (LAE) and chlorine was tested. PAA at 100 ppm and chlorine at 5 ppm showed total inactivation of the bacteriophages, while CPC at 1% and LAE at 200–500 ppm caused very little change in the bacteriophage numbers. CPC, LAE and bacteriophage were then applied to artificially *Salmonella*-contaminated chicken breasts. Breasts treated with phage only showed a 1.1 log unit reduction after 7 days, while a solution of 0.6% CPC caused a 0.9 log unit reduction and a 200 ppm LAE solution caused a 0.8 log unit reduction. The highest reduction, of 1.4 log units, was achieved by a combination of bacteriophage (9 log PFU/mL) and 0.6% CPC. When bacteriophage was applied in sequence with chemical methods to chicken skin samples, a slightly different result was achieved. Chicken skins samples immersed in chlorine at 30 ppm and then treated with bacteriophage caused a reduction by 1.8 log units, while chlorine immersion followed by distilled water treatment caused a reduction by only 0.6 log units. The highest reduction achieved in this part of the experiment was achieved by first immersing the chicken skin in 400 ppm PAA and then treating with bacteriophage, this yielded a reduction by 2.5 log units. These results highlight the effectiveness of a chemical dip application followed by a surface phage treatment in the reduction of *Salmonella*, and how phage can be used as a processing aid in a hurdle concept [105,106].

Fiorentine et al. [107] used chicken thighs and drum sticks to investigate whether the populations of *S*. *entertitids* could be reduced by bacteriophages. The chicken pieces were immersed in *S. enteritidis* phage type 4 (SE PT4) after slaughter, and then in a solution containing three types of strictly lytic phages isolated from free range chicken feces 24 h later. The pieces were stored at 5 °C and *Salmonella* numeration was conducted every 72 h. The *Salmonella* counts dropped by a multiple of 4.5 times 9 days post-treatment. 

Duc et al. [108] carried out a study whereby five lytic phages isolated from chicken skin and gizzard were used to reduce *S. enteritidis* and *S. typhimurium* on raw chicken breast incubated at 8 °C and at 25 °C. At 8 °C the phages reduced by 1.4 and 1.8 log CFU/piece for *S. enteritidis* and *S. typhimurium*, respectively, while at 25 °C reductions were 3.1 and 2.2 log CFU/piece. This shows that, at optimal conditions, the bacterial host will replicate faster, which increases phage replication [108,109]. 

Atterbury et al. [110] treated *S. typhimurium* and *S. enteritidis* contaminated chicken skins with phages Tϕ7 and Eϕ15, respectively. The skins were taken from infected Ross broiler chickens seven days post infection. After treatment with the phages, there was 1.38 log unit reduction of *S. enteritidis* and a 1.83 log unit reduction of *S. typhimurium*. 

Abhisingha et al. [111] carried out a similar study, but instead investigated the efficacy of phage during cold and freezing storage. Chicken breast was artificially contaminated with *S. typhimurium*, treated with a phage cocktail and stored at 4 °C and −20 °C. After 72 h, the breast stored at 4 °C showed reduction 0.4–1 log CFU/cm^2^ while the breast stored at −20 °C for 24 h showed 0.4–0.7 log CFU/cm^2^ reduction. This study highlighted that phage could control *Salmonella* growth effectively at 4 °C but will only be effective for the first few hours at −20 °C [111]. 

Brenner et al. [112] successfully created a phage cocktail for potential poultry industrial application by screening 78 lytic phages for efficacy against all *S. enterica* serovars linked to poultry. Of the 78 phages screened, three (which were isolated from sewage) showed a broad host range and were selected for the cocktail (SE4, SE13 and SE20). This study highlights that suitable phage cocktails can be manufactured quickly and efficiently to substitute antibiotic use. It also highlights the aspect that phage commercial cocktails can be continuously improved to ensure a broad host-range, covering the rapidly mutating *Salmonella* spp. 

Furthermore, phages can be used not only in the reduction of *Salmonella*, but also in the industrial rapid detection of *Salmonella.* This was demonstrated by Nguyen et al. [113] by using luciferase reporter phages (LRP). LRPs are genetically engineered (by including genes that code for luciferase in deep sea shrimp) to produce a bioluminescent response when the recombinant LRPs infect the *Salmonella* host.

Another exciting avenue in phage application is the use of polyvalent phages. Gambino et al. [114] discusses how polyvalent phage S144 can lyse both *Salmonella enterica* and *Cronobacter sakazakii* cells. This shows great potential for a multipathogen control using a single phage or cocktail of phages. 

Phage application shows exciting potential as an effective processing aid to reduce (and detect) *Salmonella* on chicken meat however, to ensure high efficacy, it is important to consider the extrinsic parameters such as—but not limited to—chemicals, temperature, and diffusion volume [109,110].

#### Phage Limitations and Considerations 

The largest limiting factor for the use of phage application is the efficacy; many studies show that there is an initial reduction of bacteria but no further reduction afterwards, highlighting that phage can reduce bacteria but not eradicate them completely [115]. This could be due to the phages being unable to reach and invade bacteria postprogeny, highlighting the importance of sufficient moisture to allow for diffusion of phages [116]. 

It is also important to ensure that the concentration of phages is sufficient to increase the probability of the phage and the bacteria meeting without compromising on cost implication, as it is more expensive [117,118]. Although a tempting idea, it is important to avoid recycling phage/bacteria solutions on areas where the target bacteria are prominent or exist in reservoirs. This is to avoid development of resistance to the bacteriophages [103]. 

The efficacy of phage treatment is dependent on the state of the host—if the host is replicating faster, the phage infection and progeny rate is even more rapid [104]. 

Phages are host specific, meaning that other pathogens that are not targeted by the phage are still a threat and, thus, phages cannot replace good hygiene and handling practices [115].

Although resistance to lytic phages is rare, it is still a point of consideration. Resistance may develop after continuous exposure; whereby the selective pressure of the phage may advocate for resistant properties of the bacteria [119]. This highlights the importance of legislation and the need for organizations to monitor the use of phages to ensure they is used in such a manner that prevents instances of this nature as far as possible.

The permission for phage use differs from country to country. Phage application as a processing aid is permitted in the USA, Canada Switzerland, New Zealand, Australia, and Israel. In the EU, however, it is only allowed in the Netherlands and is not included on the qualified presumption of safety (QPS) list [120,121]. 

Due to the ability of various chemicals to influence the stability and efficacy of phages, as well the legislative aspects to consider, the industry requires some careful planning for successful implementation with other chemical interventions [105].

## 6. Concluding Remarks

Global poultry meat consumption is increasing due to the perceived health benefits and few religious associations with white meat. As raw chicken is a good reservoir for *Salmonella*, the increase in poultry meat production and consumption has been accompanied by a spike in foodborne illness due to *Salmonella* infection traced back to poultry meat products.

There are several current control methods for *Salmonella* in the processing setting, namely, good hygiene practices and the use of chlorine containing compounds (examples include: chlorine dioxide and sodium chlorite) as well as organic acids (such as lactic acid and succinic acid). Although chlorine has been shown to have a high efficacy, it has also been shown that it places immense selective pressure on *Salmonella* spp. and is not sustainable in the long term if antimicrobial resistant spp. are to be avoided. Furthermore, chlorine is prohibited for use in many countries due to the high rate of resistance shown by *Salmonella* to chlorine containing products. Similarly, organic acids are effective in the reduction of *Salmonella* on chicken, but at higher concentrations organic acids may influence the sensory properties of the meat.

Thus, phages show great potential as a new control measure in the processing setting. Phages show great promise in being integrated into the large scale hurdle concept of an industrial chicken processing setting. Phage use, however, requires careful consideration for things such as other chlorine (to avoid inactivation) and sufficient liquid for diffusion. Still, phages are preferable due to their ability to place pressure on a single target organism as opposed to a whole microbial community, decreasing the magnitude of selective pressure that is seen with chemical use.

## Figures and Tables

**Table 1:** Comparison of Salmonella food category pairs and number of outbreaks resulting in illness [20].

Food Category	No. Outbreaks	No. Illness
Turkey	2	580
Chicken	11	299
Fruits	10	421
Other	1	199
Vegetable row crops	2	178

**Table 2:** Summary of GHP and various control measures to consider when slaughtering broiler chickens to reduce the risk of *Salmonella* in the final chicken meat product (adapted from [31]).

**Slaughter Practices**		
1.	Carcass dressing	Continuous stream of clean water for washing If carcass is seen to have excessive feces it should be thrown away Chemicals may be used during this step for decontamination; these should be approved by authorities
2.	Scald	Water with a flow that is counter current, rapid and continuously mixed should be used Appropriate temperature and pH (by addition of approved chemicals) should be used to reduce *Salmonella* Sufficient and regular cleaning of scalding tanks and good waste-water management
3.	Defeather	Chickens should have had appropriate length of time for feed withdrawal to avoid contamination during defeathering Avoid accumulation of feathers on machinery Appropriate cleaning, sanitizing and maintenance of machinery and with emphasis on rubber fingers
4.	Pull off head	Any drip from the crop or rupturing of the crop should be averted; this is performed by pulling the head in the downward direction
5.	Re-hang carcass	Rehanging of carcasses should be performed by personnel and not automatically to avoid contamination Corrective action should be in place for carcasses that are dropped onto the floor
6.	Eviscerate	Rupturing viscera can be avoided by processing birds of the same size, this also requires regular adjustment to equipment
7.	Remove crop	Should be removed in such a way so as to avoid contamination of the carcass A chlorine solution or Tri Sodium Phosphate (TSP) dip may be applied at this step, just after the carcass has been defeathered and eviscerated to reduce *Salmonella*
8.	Removal of neck skin	Should be removed in such a way so as to avoid contamination of the carcass
**Prepackaging Practices**		
9.	Inside–outside washing of carcass	Interior and exterior of carcass should be cleaned extensively using high-pressure chlorinated water stream as well as to reduce *Salmonella* The use of brushes may be utilized for inside–outside washing to assist in removal of evident contamination
10.	Extra wash step	Acidified Sodium Chlorite (ASC), or TSP may be applied at this step via spray or dip to reduce *Salmonella*
11.	Postmortem analysis	Analysis should be conducted with sufficient time and lighting to clearly see any contamination, carcass defects, or damage
12.	Chilling (dip)	Rapid chilling is advised to inhibit growth of spoilage microorganisms and pathogens Important that whole carcass is cooled to desired temperature by the end of the chilling step If a dip application is utilized for chilling, chemicals may be added to reduce *Salmonella*, such as chlorine or oxygen composites and organic acids. Sufficient time should be allowed for this liquid to drip off of carcass postapplication to reduce contamination further down the line It is important that flow of water is counter current, rapid and continuous
13.	Additional dip	Once carcass cooled an additional cooled dip containing ASC or chlorine may further reduce *Salmonella*
14.	Portioning	Carcasses should remain at low temperatures and be portioned swiftly after chilling
15.	Packaging of portions/whole carcass	Packaging should not leak any fluid from chicken to prevent contamination Clear instructions for cooking, storage and handling according to regulations should be visible for consumer Carcasses should remain at low temperatures Use of irradiation may be used at this step to further reduce *Salmonella*
**Post-Packaging and Transport Practices**		
16.	Chilling/freezing	Desired temperature should be uniform throughout carcass at end of chilling step
17.	Storage	Important to keep carcasses at low temperature to inhibit *Salmonella* growth
18.	Transporting	Same as step 17
19.	Store/consumer	Same as step 17

**Table 3:** Parameters for survival and growth of *Salmonella* (adapted from [34,41]).

Parameter	Approximate Growth Range
Temperature	5–46 °C (optimum = 38 °C)
Water activity	0.94–0.99
pH	3.8–9.5

**Table 4:** Some of the widely used safe and suitable antimicrobials stipulated for use in poultry processing to produce raw poultry meat products in the United States (data from [50]).

Antimicrobial	Product	Amount
Aqueous sulfuric acid/sodium sulfate	Wash, spray or immersion dip on surface of poultry products	Concentration that employs pH of 1–2.2 of poultry Measured on the meat surface
Acidified sodium chlorite	Poultry pieces and carcasses	500–1200 ppm. May be used in a mixture with any “generally recognized as safe” (GRAS) acid to obtain pH 2.3–2.9
	Poultry carcasses, pieces, organs and trimmings	May be added to a GRAS acid to obtain pH 2.2–3 May be further diluted with basic sodium bicarbonate to obtain pH 5–7.5 Use in a dip/spray, should not have sodium chlorite concentration >1200 mg/kg or chlorine dioxide concentration >30 mg/kg Use in a prechilling or chilling solution for carcasses, sodium chlorite should be 50–150 ppm Contact time is not detrimental as long as temperature is 0–15 °C
Bacteriophage solution (Salmonella specific)	Applied to feathers of live poultry preslaughter	Spray or fine mist application, or wash
Calcium hypochlorite	Used on eviscerated or whole chicken carcass	Spray application should not have free available chlorine >50 ppm
	Water used for poultry processing and for chiller water	Free available chlorine should not be >50 ppm for inlet water Measure at potable water inlet
	Water recirculated from chiller via heat exchangers	Free available chlorine should not be >5 ppm at inlet to chiller
	Retreating carcasses that are contaminated	Free available chlorine should be 20–50 ppm
	Giblets	Free available chlorine should not be >50 ppm at inlet to chiller
Chlorine gas	Used on carcass that is whole or has been eviscerated	Spray application where free available chlorine should not >5 ppm Measured before application
	Used in water of chiller	Free available chlorine should not >50 ppm Should be measured at inlet of potable water
	Water recirculated from chiller via heat exchangers	Free available chlorine should not >5 ppm Measured at chiller inlet
	Retreating carcasses that are contaminated	Free available chlorine should be 20–50 ppm
	Giblets	Free available chlorine should not >50 ppm. Measured at inlet to chiller
Chlorine dioxide	Water used for processing of poultry	Residual chlorine dioxide should not >3 ppm
DBDMH (1,3-dibromo-5,5- dimethylhydantoin)	Used in water of chiller and water of inside–outside bird washer (IOBW). In addition, used for processing of poultry carcasses, organs and pieces.	Active bromine should not be >100 ppm
	Added to water for ice making which is then used in processing of poultry	Active bromine should not >100 ppm (or max 90 mg DBDMH per kg water)
Hypochlorous acid	Used on carcass that is whole or has been eviscerated	For spray application, free available chlorine should not >50 ppm Measured before application
	Added to water used for processing of poultry	Free available chlorine should not >50 ppm
	Used in water for chiller	Free available chlorine should not >50 ppm Measured at inlet of potable water
	Water recirculated from chiller via heat exchangers	Free available chlorine should not >5 ppm Measure at chiller inlet
	Used for re-treating poultry carcasses that are contaminated	Free available chlorine should be 20–50 ppm
	Giblets	Free available chlorine should not >50 ppm
Citric and Hydrochloric acid solution (pH 1–2)	Poultry carcasses, pieces, organs and trimmings	Spray or dip application with 2–5 s contact time Measure before application
1.87% citric acid, 1.72% phosphoric acid and 0.8% hydrochloric acid solution	Poultry carcasses	Spray application with 1–2 s contact time. Should run off carcasses for 30 s
Lactic acid	Poultry carcasses, pieces, organs and trimmings	5% concentration for post chilling
Peroxyacetic acid (PAA), hydrogen peroxide (HP), acetic acid (AA), and 1 hydroxyethylidene-1, 1 diphosphonic acid (HEDP) solution	Used in water for poultry processing, scalding tanks, ice production and spray applications	PAA should not >220 ppm, HP should not >110 ppm, HEDP should not >13 ppm
PAA, octanoic acid (OA), Peroxyoactanoic acid (POA) HP, AA, HEDP solution	Carcasses, pieces, trimmings and organs	PAA should not >220 ppm, HP should not >110 ppm, HEDP should not >13 ppm
PAA, HP, HEDP solution	Added to water for processing of carcasses and pieces. Applied via spray, dip, wash or added to chiller or scalding tank.	PAA should not >2000 ppm and HEDP should not >136 ppm
PAA, HP, AA, HEDP solution	Used in water or ice for applied on whole carcasses, pieces, trimmings and organs. Applied via spray, dip, wash or added into chiller or scalding tank water	PAA should not >220 ppm, HP should not >80 ppm, HEDP should not exceed 1.5 ppm
	Added to process water for application to carcasses, pieces, trimmings and organs via spray application, dip, rinse, wash or added into chiller or scalding tank water	PAA should not >2020 ppm, HP should not exceed 160 ppm, HEDP should not exceed 11 ppm
Sodium hypochlorite	Applied to eviscerated or whole carcasses	For spray application, free available chlorine should not >50 ppm
	Added to water for processing of poultry	Free available chlorine should not >50 ppm at potable water inlet
	Added to water in chiller	should not >50 ppm
	Added to water recirculated from chiller via heat exchangers	Free available chlorine should not >5 ppm at inlet to chiller
	Retreatment of contaminated carcasses	Free available chlorine should be 20–50 ppm
	Giblets	Free available chlorine should be 20–50 ppm

**Table 5:** Various treatment methods of *S*. *enteritidis* on chicken skin and the resulting reductions (adapted from [104]).

Treatment	Concentration	Time	Reduction (log CFU/cm^2^)
Control	N/A		0
Water	N/A	30 min	0.2
Dichloroisocyanurate	200 ppm	10 min	0.8
Peroxyacetic acid	100 ppm	10 min	0.8
Lactic acid	2%	90 s	0.8
Bacteriophage	10^9^ PFU/ml	30 min	1
